# Valor Prognóstico de Rigidez Aórtica usando Ressonância Magnética Cardiovascular em Idosos com Suspeita ou Confirmação de Doença Arterial Coronariana

**DOI:** 10.36660/abc.20210452

**Published:** 2022-05-04

**Authors:** Yodying Kaolawanich, Thananya Boonyasirinant

**Affiliations:** 1 Department of Medicine Faculty of Medicine Siriraj Hospital Mahidol University Bangkok Tailândia Division of Cardiology, Department of Medicine – Faculty of Medicine Siriraj Hospital, Mahidol University, Bangkok – Tailândia

**Keywords:** Rigidez Aórtica, Ressonância Magnética Cardiovascular, Doença arterial Coronariana, Idoso, Prognóstico

## Abstract

**Fundamento:**

A rigidez aórtica é considerada um marcador de doença cardiovascular. A ressonância magnética cardiovascular (RMC) permite realizar uma avaliação abrangente da rigidez aórtica e da isquemia miocárdica em um único exame. Entretanto, dados prognósticos relacionados à rigidez aórtica em pacientes idosos permanecem limitados.

**Objetivo:**

Determinar o valor prognóstico da rigidez aórtica usando a velocidade da onda de pulso (VOP) baseada em RMC em pacientes idosos com doença arterial coronariana (DAC).

**Métodos:**

Foram cadastrados pacientes consecutivos com idade >70 com indicação para RMC com perfusão de estresse com adenosina incluindo VOP, entre 2010 e 2014. Os pacientes foram acompanhados para verificar a ocorrência de eventos cardíacos adversos maiores (MACE), incluindo mortalidade cardíaca, infarto do miocárdio não fatal, hospitalização por insuficiência cardíaca, revascularização tardia (>180 dias após a RMC), e acidente vascular isquêmico. Foram realizadas análises univariadas e multivariadas para determinar os preditores de MACE. Um p-valor <0,05 foi considerado estatisticamente significativo.

**Resultados:**

A VOP média foi 13,98±9,00 m/s. Depois de um período mediano de acompanhamento de 59,6 meses em 263 pacientes (55% do sexo feminino, 77±5 anos), ocorreram 61 MACE. Pacientes com VOP elevada (>13,98 m/s) tiveram índices de MACE significativamente mais altos (FC 1,75; IC 95% 1,05-2,94; p=0,03) que os dos pacientes com VOP não elevada (<13,98 m/s). A análise multivariada demonstrou que pressão arterial diastólica, fração de ejeção ventricular esquerda (FEVE), isquemia miocárdica, e VOP elevada são preditores independentes de MACE (p<0,05 para todos). A VOP apresentou um valor prognóstico incremental em relação a dados clínicos, FEVE e isquemia (qui-quadrado global aumentado = 7,25, p=0,01).

**Conclusão:**

A rigidez aórtica, usando-se a RMC, é um preditor independente forte de eventos cardiovasculares em pacientes idosos com suspeita de DAC ou DAC confirmada.

## Introdução

A rigidez arterial aumenta com a idade como preditor independente de eventos cardiovasculares, incluindo a mortalidade.^1 - 4^ Há várias maneiras de se medir a rigidez arterial, tais como, ultrassonografia, tonômetro carótido-femoral, e ressonância magnética cardiovascular (RMC). A medição da velocidade da onda de pulso (VOP) aórtica por tonometria já foi amplamente utilizada. Entretanto, a RMC é geralmente o método preferido. Medições de VOP baseadas em RMC já foram bastante validadas (comparadas com registros de pressão invasivos) com grande reprodutibilidade.^5^ Os benefícios da RMC incluem a obtenção de imagens de cortes transversais cobrindo o comprimento aórtico desejado, alta resolução espacial, e medição direta do comprimento aórtico sem presumir a distância (ao contrário da tonometria), sem radiação ionizante.

A idade avançada é um dos marcadores de doença cardiovascular (DCV) mais importantes, incluindo a doença arterial coronariana (DAC). As DCV são responsáveis por mais de 80% das mortes de indivíduos com 65 anos de idade ou mais em países desenvolvidos.^6^ Portanto, é fundamental fazer o diagnóstico e a estratificação de risco de DAC em pacientes idosos. A RMC oferece uma avaliação abrangente de DAC com precisão muito alta.^7^ Além disso, a RMC com estresse com adenosina apresenta fortes evidências para o prognóstico de eventos cardiovasculares futuros em pacientes com suspeita de DAC ou DAC confirmada.^8^ Dados anteriores indicam que a RMC com estresse realizada em idosos deambulantes é segura e bem tolerada.^9 , 10^ A RMC pode avaliar a VOP e realizar um teste de estresse em um único exame. Recentemente demonstramos a associação entre rigidez aórtica e isquemia miocárdica, bem como o valor prognóstico da rigidez aórtica utilizando-se a RMC.^11 , 12^ Entretanto, há dados limitados em relação ao prognóstico de VOP pela RMC em pacientes idosos.

O objetivo do estudo era determinar o valor prognóstico da VOP em termos de eventos cardíacos adversos maiores (MACE) em pacientes idosos com suspeita de DAC ou DAC confirmada.

## Métodos

### População do estudo

Este estudo cadastrou pacientes consecutivos acima de 70 anos de idade com suspeita de DAC ou DAC confirmada que foram encaminhados para RMC com estresse por adenosina de outubro de 2010 a fevereiro de 2014 em nossa clínica ambulatorial. Em nossa instituição, a medição da rigidez aórtica utilizando a VOP foi incorporada à rotina do protocolo de RMC abrangente para avaliação de DAC. O histórico médico detalhado foi obtido no dia do estudo por RMC.

Os critérios de exclusão incluíram (1) exame de RMC incompleto, (2) contraindicações à RMC (por exemplo, marca-passo) ou adenosina (por exemplo, bloqueio atrioventricular de alto grau), (3) condição clínica instável, (4) pacientes com doenças aórticas envolvendo medições de VOP (por exemplo, um aneurisma aórtico^13^ ), (5) má qualidade da imagem da RMC, e (6) pacientes sem dados de acompanhamento. Pacientes com taxa de filtração glomerular de <30 ml/min/1,73 m^2^ no período de 30 dias antes da RMC também foram excluídos.

O comitê de ética institucional aprovou este estudo retrospectivo e dispensou a necessidade de consentimento informado por escrito adicional.

## Protocolo da RMC ( Materiais suplementares )

### Análises de imagens de RTG, perfusão e cine ( Materiais suplementares )

#### Análise de VOP11

Foi aplicado um software dedicado de imagem cardiovascular para a análise de VOP, realizada independentemente do estudo de perfusão de RTG. Os contornos da porção ascendente e da porção descendente da aorta torácica foram desenhados manualmente para obter o fluxo (m/s) nos dois locais durante todas as fases do ciclo cardíaco. A curva fluxo-tempo correspondente foi gerada. O tempo de chegada da onda de pulso foi medido como ponto de interceptação da extrapolação linear da linha de base e do pico sistólico precoce, enquanto o comprimento do trajeto aórtico foi determinado pela reconstrução multiplanar da aquisição de meia-Fourier axial a partir de imagem em equilíbrio estável. A vista sagital reconstruída do comprimento do trajeto foi mostrada como a linha de centro dos níveis da porção ascendente à porção descendente da aorta torácica, correspondendo ao mesmo nível obtido na RMCR-VP.^11^

A VOP entre a porção ascendente e a porção descendente da aorta torácica foi calculada como:


VOP=Δx/ΔT(m/s)

Em que Δ x reflete o comprimento do trajeto aórtico entre a porção ascendente e a porção descendente da aorta torácica, e Δ T representa o intervalo de tempo entre a chegada do pé da onda de pulso nesses dois níveis correspondentes ( Figura suplementar 1 ).

#### Variabilidade da medição de VOP intraobservador e interobservador

Aproximadamente 10% da coorte do estudo foram selecionados aleatoriamente, usando um gerador de número aleatório no Microsoft Excel versão 2016, para medir a variabilidade do primeiro observador 4 semanas após a análise inicial, e a variabilidade do segundo observador independente, cego em relação aos resultados iniciais.

#### Acompanhamento clínico

Foram coletados dados de acompanhamento de consultas clínicas e prontuários médicos. A adjudicação do evento foi cega em relação a dados clínicos e da RMC. Os pacientes foram acompanhados para verificar a ocorrência de MACE, definidos como resultados compostos de mortalidade cardíaca, infarto do miocárdio (IM) não fatal, hospitalização por insuficiência cardíaca, revascularização tardia (>180 dias após a RMC), e acidente vascular isquêmico. A necessidade de terapia de revascularização em até 180 dias após a RMC foi considerada consequência dos resultados da RMC, e, portanto, excluída da análise.

## Análise estatística

As análises estatísticas foram realizadas utilizando-se o software IBM SPSS Statistics for Windows versão 20.0 (IBM Corp., Armonk, NY, EUA). Variáveis contínuas com distribuição normal foram expressas como médias ± desvio padrão (DP), e variáveis contínuas com distribuição não normal foram apresentadas como mediana e faixa interquartil. A distribuição de normalidade das variáveis foi examinada pelo teste de Kolmogorov-Smirnov. As variáveis categóricas foram apresentadas como números absolutos e porcentagens. Os pacientes foram divididos em dois grupos, com base em seus valores de VOP. Os grupos eram os de VOP elevada e de VOP não elevada, usando-se o valor da média de VOP de todos os pacientes como nível de corte. As variabilidades intraobservador e interobservador das medições de VOP foram expressas como coeficiente de correlação intraclasse (ICC), intervalo de confiança (IC) de 95% e viés de ±2 DP (para limites de concordância) usando-se a análise de Bland-Altman.

Diferenças entre pacientes com VOP elevada e não elevada, bem como entre pacientes com ou sem MACE, foram comparadas usando-se o teste t de Student não pareado, ou o teste U de Mann-Whitney para variáveis contínuas, e o teste qui-quadrado ou teste exato de Fisher para variáveis categóricas, conforme apropriado.

Resultados compostos entre os dois grupos foram estimados usando-se o método de Kaplan-Meier e comparados com o teste de Log-rank. Para analisar os preditores de MACE, uma análise de regressão de Cox foi realizada para avaliar preditores univariados. Variáveis (características de linha de base, medicamentos no momento da RMC, e parâmetros da RMC) com p-valor <0,05 na análise univariada foram incluídos para análise multivariada usando o método ENTER. Uma análise de característica de operação do receptor (ROC) foi usada para determinar o melhor valor de VOP preditor de MACE.

Para avaliar o valor prognóstico incremental dos preditores significativos, valores globais de qui-quadrado foram calculados após somar preditores na seguinte ordem: clínicos, FEVE, isquemia miocárdica e VOP.

Todos os testes estatísticos foram bicaudais, enquanto todos os p-valores menores que 0,05 foram considerados indicativos de significância estatística.

## Resultados

### Características do paciente

Foram cadastrados 269 pacientes, sendo que dois foram excluídos por terem aneurisma aórtico, e quatro foram excluídos devido a perda de dados de acompanhamento. Nenhum paciente foi excluído devido à má qualidade da imagem, e 263 foram incluídos na análise final. A idade média foi de 77,3±5,2 anos. A Tabela 1 resume os dados clínicos dos pacientes. Duzentos e oito pacientes foram encaminhados para o primeiro diagnóstico de DAC. Cinquenta e cinco tinham sido diagnosticados com DAC, incluindo 4 com IM anterior documentado. Em geral, a coorte do estudo teve uma FEVE média de 68,1±15,1%. Isquemia miocárdica foi detectada em 95 (36,1%) pacientes. Trinta e nove (14,8%) tinham RTG, e todos apresentaram um padrão de DAC (RTG subendocárdico ou transmural). Nenhum paciente apresentou frequência cardíaca irregular (como fibrilação atrial) durante a aquisição da VOP. A VOP média foi 13,98±9,00 m/s. Histórico de hipertensão, diabetes mellitus, e pressão arterial sistólica foram preditores independentes de VOP elevada (>13,98 m/s) ( Tabela 2 ).

**Tabela 1:** – Características clínicas de pacientes com e sem VOP elevada

	Total (n=263)	VOP elevada (n=83)	VOP não elevada (n=180)	p-valor
Idade (anos)	77,3±5,2	77,9±5,1	77,1±5,2	0,19
Feminino	144 (54,8)	50 (60,2)	94 (52,2)	0,23
Índice de massa corporal (kg/m^2^)	26,3±4,1	26±4,1	26,4±4,2	0,49
PA sistólica (mmHg)	139,3±19,7	144,7±18,1	136,9±19,9	**0,003**
PA diastólica (mmHg)	70,5±11,1	71,4±11,3	70,2±10,9	0,42
Frequência cardíaca (pulsos/minuto)	76,6±13,9	76,6±15,5	76,6±13,2	0,99
**Histórico clínico**				
Hipertensão	235 (89,4)	81 (97,6)	154 (85,6)	**0,01**
Diabetes mellitus	145 (55,1)	58 (69,9)	87 (48,3)	**0,001**
Hiperlipidemia	197 (74,9)	60 (72,3)	137 (76,1)	0,51
Doença arterial coronariana	55 (20,9)	20 (24,1)	35 (19,4)	0,39
Revascularização prévia	12 (4,6)	4 (4,8)	8 (4,4)	0,89
Acidente vascular isquêmico	13 (4,9)	3 (3,6)	10 (5,6)	0,50
Fumante	28 (10,6)	7 (8,4)	21 (11,7)	0,43
**Medicamentos**				
IECA ou BRA	130 (49,4)	43 (51,8)	87 (48,3)	0,60
Aspirina	134 (50,9)	45 (54,2)	89 (49,4)	0,47
Betabloqueador	124 (47,2)	38 (45,8)	86 (47,8)	0,76
Bloqueador dos canais de cálcio	96 (36,5)	27 (32,5)	69 (38,3)	0,36
Estatina	147 (55,9)	50 (60,2)	97 (53,9)	0,34
**RMC**				
Massa de VE (g/m^2^)	84,3±24,5	83,6±25,5	84,7±24,0	0,74
Fração de ejeção VE (%)	68,1±15,1	69,8±14,1	67,3±15,5	0,21
Isquemia miocárdica	95 (36,1)	28 (33,7)	67 (37,2)	0,58
Realce tardio pelo gadolínio	39 (14,8)	15 (18,1)	24 (13,3)	0,32
VOP (m/s)	13,98±9,00	22,09±12,28	10,24±2,22	**<0,001**

*Os valores são números (porcentagens) ou média ± DP. Os valores em ***negrito*** são <0,05. IECA: inibidor da enzima de conversão da angiotensina; BRA: bloqueador de receptores da angiotensina II; PA: pressão arterial; RMC: ressonância magnética cardiovascular; VE: ventrículo esquerdo; VOP: velocidade de onda de pulso; DP: desvio padrão.*

**Tabela 2:** – Preditores de VOP elevada (>13,98 m/s)

	Análise univariada		Análise multivariada	

	RC (IC 95%)	p-Valor	RC (IC 95%)	p-Valor
Idade (anos)	1,03 (0,98, 1,09)	0,19		
Feminino	1,39 (0,82, 2,35)	0,23		
Índice de massa corporal (kg/m^2^)	0,98 (0,92, 1,04)	0,49		
PA sistólica (por 10 mmHg)	1,23 (1,07, 1,41)	**0,003**	1,23 (1,07, 1,42)	**0,01**
PA diastólica (por 10 mmHg)	1,10 (0,87, 1,40)	0,42		
Hipertensão	6,84 (1,58, 29,54)	**0,01**	6,06 (1,36, 26,97)	**0,02**
Diabetes mellitus	2,48 (1,43, 4,31)	**0,001**	2,09 (1,18, 3,70)	**0,01**
Hiperlipidemia	0,82 (0,45, 1,48)	0,51		
Doença arterial coronariana	1,32 (0,71, 2,54)	0,39		
Revascularização prévia	1,09 (0,32, 3,72)	0,89		
Acidente vascular isquêmico	0,64 (0,17, 2,38)	0,50		
Fumante	0,70 (0,28, 1,71)	0,43		
IECA ou BRA	1,15 (0,68, 1,93)	0,60		
Aspirina	1,21 (0,72, 2,04)	0,47		
Betabloqueador	0,92 (0,55, 1,56)	0,76		
Bloqueador dos canais de cálcio	0,78 (0,45, 1,34)	0,36		
Estatina	1,30 (0,76, 2,20)	0,34		
Massa de VE (g/m^2^)	0,99 (0,98, 1,01)	0,74		
Fração de ejeção VE (por 10%)	1,13 (0,94, 1,35)	0,21		
Isquemia miocárdica	0,86 (0,50, 1,48)	0,58		
Realce tardio pelo gadolínio	1,43 (0,71, 2,90)	0,32		

*Os valores em ***negrito*** são <0,05. IECA: inibidor da enzima de conversão da angiotensina; BRA: bloqueador de receptores da angiotensina II; PA: pressão arterial; RMC: ressonância magnética cardiovascular; VE: ventrículo esquerdo; VOP: velocidade de onda de pulso; IC: intervalo de confiança; RC: razão de chance.*

#### Variabilidade intraobservador e interobservador na medição de VOP

**Figura 1:**
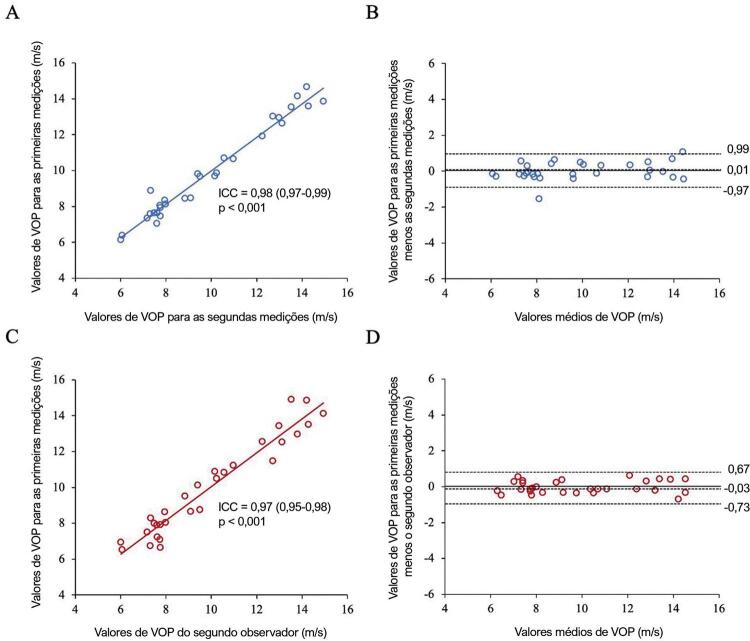
– Variabilidade intraobservador e interobservador de medições de VOP. Correlação intraclasse (A para intraobservador e C para interobservador) e gráfico de Bland-Altman (B para intraobservador e D para interobservador). ICC: coeficiente de correlação intraclasse; VOP: velocidade de onda de pulso.

Houve menos variabilidade intraobservador e interobservador nas medições de VOP por RMC-VP ( Figura 1 ). Para os 30 pacientes selecionados aleatoriamente, os valores médios de VOP±DP foram 9,88±2,73 m/s e 9,87±2,59 m/s para o primeiro observador na análise inicial e 4 semanas depois, respectivamente, e 9,94±2,67 m/s para o segundo observador na análise inicial. Não houve viés significativo (diferença média para intraobservador =0,01±0,49 m/s, p=0,98 e para interobservador =-0,03±0,35 m/s, p=0,93) ( Figura 1B e 1D , respectivamente).

#### Resultados principais: MACE

Durante o período mediano de acompanhamento de 59,6 meses (faixa interquartil: 36,6, 68,2 meses), ocorreram 61 MACE. As características clínicas incluindo variáveis de RMC de pacientes com e sem MACE são apresentadas na Tabela suplementar 1 . Os pacientes com MACE tinham pressão arterial diastólica significativamente mais baixa, massa de VE mais alta, FEVE mais baixa, e maior prevalência de isquemia e RTG.

**Figura 2:**
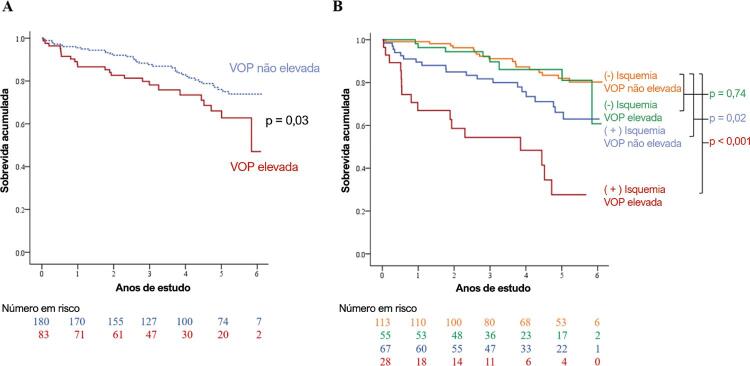
– Curvas de Kaplan-Meier for MACE. Para toda a coorte, pacientes com VOP elevada tiveram índices de MACE significativamente mais altos que os dos pacientes com VOP não elevada (Figura 2A). A Figura 2B apresenta as curvas de Kaplan-Meier estratificadas pela presença de isquemia e sem VOP elevada. MACE: eventos cardiovasculares adversos maiores; VOP: velocidade de onda de pulso.

A Tabela 3 demonstra os eventos cardiovasculares na coorte do estudo. A Figura 2A apresenta as curvas de Kaplan-Meier de pacientes com e sem VOP elevada. Pacientes com VOP elevada tiveram índices de MACE significativamente mais altos que os dos pacientes com VOP não elevada. A Figura 2B apresenta as curvas de Kaplan-Meier estratificadas pela presença de isquemia e sem VOP elevada. Pacientes com VOP não elevada e isquemia negativa tiveram os melhores resultados, enquanto pacientes com VOP elevada e isquemia positiva tiveram os piores resultados. Observe que pacientes com VOP não elevada e isquemia positiva não tiveram diferença no índice de MACE se comparados a pacientes com VOP elevada e isquemia negativa (FC 2,03, IC 95% 0,89-4,63, p=0,09).

**Tabela 3:** – Eventos cardiovasculares

	Total (n=263)	VOP elevada (n=83)	VOP não elevada (n=180)	RC (IC 95%)	p-valor
MACE^a^	61 (23,2)	24 (28,9)	37 (20,6)	1,75 (1,05, 2,94)	**0,03**
Mortalidade cardíaca	5 (1,9)	2 (2,4)	3 (1,7)	1,68 (0,28, 10,07)	0,57
Infarto do miocárdio não fatal	24 (9,1)	9 (10,8)	15 (8,3)	1,60 (0,70, 3,67)	0,27
Hospitalização por insuficiência cardíaca	36 (13,7)	15 (18,1)	21 (11,7)	1,94 (0,99, 3,81)	0,05
Revascularização coronária tardia	16 (6,1)	5 (6,0)	11 (6,1)	1,17 (0,41, 3,39)	0,77
Acidente vascular isquêmico	11 (4,2)	7 (8,4)	4 (2,2)	5,04 (1,47, 17,32)	**0,01**

*MACE = resultados compostos de mortalidade cardíaca, infarto do miocárdio não fatal, hospitalizados for insuficiência cardíaca, revascularização coronária tardia, e acidente vascular isquêmico. ^a^ Dezenove pacientes tiveram mais de um evento. Os valores são números (porcentagens). Os valores em ***negrito*** são <0,05. IC: intervalo de confiança; RC: razão de chance; MACE: eventos cardiovasculares adversos maiores; VOP: velocidade de onda de pulso.*

**Figura 3:**
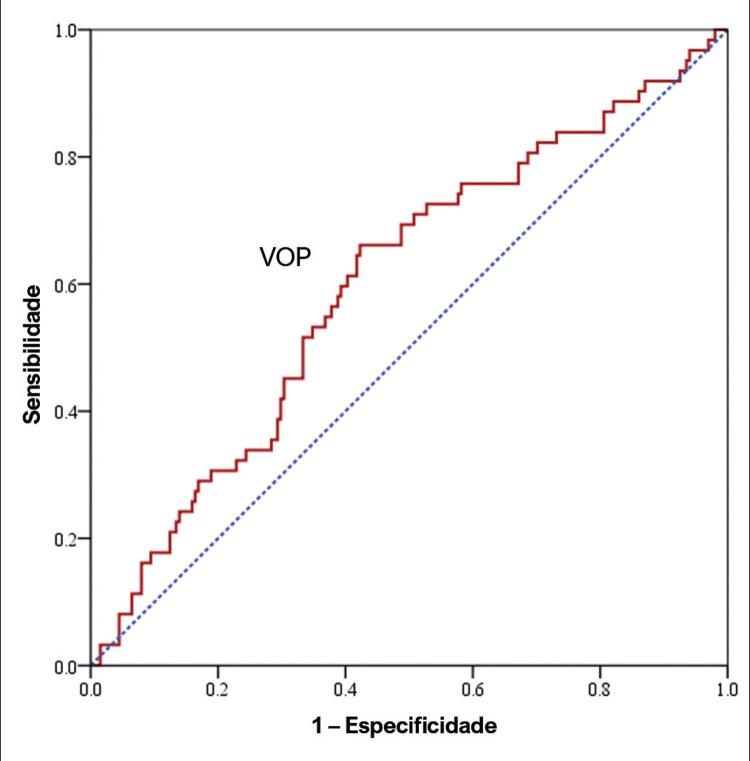
– Uma curva ROC demonstra o melhor valor de VOP para prever MACE. MACE: eventos cardiovasculares adversos maiores; VOP: velocidade de onda de pulso; ROC: característica de operação do recebedor.

Uma curva ROC ( Figura 3 ) demonstrou o melhor valor de VOP de 11,16 m/s para prever MACE com uma sensibilidade de 71% e especificidade de 50%.

Análises univariadas e multivariadas para previsão de MACE são apresentadas na Tabela 4 . A análise univariada demonstrou pressão arterial diastólica, histórico de DAC, massa no VE, FEVE, isquemia, RTG e VOP elevada como preditores. A análise multivariada revelou que pressão arterial diastólica, FEVE, isquemia, e VOP elevada são preditores independentes de MACE.

**Tabela 4:** – Preditores de MACE

	Análise univariada		Análise multivariada	

	RC (IC 95%)	p-valor	RC (IC 95%)	p-valor
Idade (anos)	1,03 (0,98, 1,08)	0,29	1,02 (0,97, 1,08)	0,43
Feminino	1,01 (0,61, 1,67)	0,98		
Índice de massa corporal (kg/m^2^)	0,95 (0,89, 1,01)	0,08		
PA sistólica (por 10 mmHg)	0,89 (0,78, 1,02)	0,10		
PA diastólica (por 10 mmHg)	0,75 (0,59, 0,96)	**0,01**	0,76 (0,59, 0,97)	**0,03**
Hipertensão	1,49 (0,60, 3,71)	0,40		
Diabetes mellitus	1,17 (0,70, 1,94)	0,55		
Hiperlipidemia	1,37 (0,73, 2,58)	0,33		
Doença arterial coronariana	1,80 (1,02, 3,17)	**0,04**	1,25 (0,69, 2,26)	0,47
Revascularização prévia	1,56 (0,62, 3,89)	0,35		
Acidente vascular isquêmico	0,63 (0,15, 2,58)	0,52		
Fumante	1,37 (0,65, 2,88)	0,41		
IECA ou BRA	1,39 (0,84, 2,31)	0,20		
Aspirina	1,35 (0,81, 2,25)	0,25		
Betabloqueador	1,15 (0,69, 1,89)	0,60		
Bloqueador dos canais de cálcio	0,88 (0,52, 1,49)	0,62		
Estatina	0,99 (0,60, 1,64)	0,97		
Massa de VE (g/m^2^)	1,02 (1,01, 1,03)	**0,001**	1,01 (0,99, 1,02)	0,41
Fração de ejeção VE (por 10%)	0,75 (0,65, 0,86)	**<0,001**	0,84 (0,70, 0,99)	**0,04**
Isquemia miocárdica	3,10 (1,86, 5,18)	**<0,001**	2,26 (1,23, 4,14)	**0,01**
Realce tardio pelo gadolínio	2,30 (1,27, 4,19)	**0,01**	1,08 (0,55, 2,12)	0,8
VOP elevada (>13,98 m/s)	1,75 (1,05, 2,94)	**0,03**	1,99 (1,17, 3,40)	**0,01**

*Os valores em ***negrito*** são <0,05. IECA: inibidor da enzima de conversão da angiotensina; BRA: bloqueador de receptores da angiotensina II; PA: pressão arterial; VE: ventrículo esquerdo; VOP: velocidade de onda de pulso; IC: intervalo de confiança; RC: razão de chance; MACE: eventos cardiovasculares adversos maiores; VOP: velocidade de onda de pulso.*

#### Valor prognóstico incremental da VOP

A Tabela 5 mostra o valor prognóstico incremental de dados clínicos e de RMC para a previsão de MACE. Quando o prognóstico foi avaliado de maneira hierárquica (somente dados clínicos, clínicos+FEVE, clínicos+FEVE+isquemia miocárdica, e clínicos+FEVE+isquemia miocárdica+VOP), FEVE e isquemia apresentaram um valor prognóstico incremental sobre os dados clínicos. A VOP acrescentou um valor prognóstico incremental adicional sobre FEVE e isquemia.

**Tabela 5:** – Valor prognóstico incremental da VOP para MACE

	Global 𝛘^2^	Aumento em 𝛘^2^	p-valor
Clínicos	10,19	–	–
Clínicos + FEVE	27,11	14,17	**<0,001**
Clínicos + FEVE + Isquemia miocárdica	38,55	10,02	**0,01**
Clínicos + FEVE + Isquemia miocárdica + VOP	45,21	7,25	**0,01**

*Os valores em ***negrito*** são <0,05. Clínicos=idade, sexo feminino, pressão arterial diastólica, e histórico de doença arterial coronariana. 𝛘^2^ = qui-quadrado. FEVE: fração de ejeção ventricular esquerda; VOP: velocidade de onda de pulso.*

## Discussão

Os resultados mostram que a rigidez aórtica, avaliada por RMC-VP, é um forte preditor de MACE, independentemente de fatores de risco tradicionais, função cardíaca, isquemia miocárdica, e RTG, em pacientes idosos com suspeita de DAC ou DAC confirmada. A VOP também apresentou um valor prognóstico incremental em relação a dados clínicos, FEVE, e isquemia miocárdica.

### Envelhecimento e alteração vascular

O envelhecimento vascular está associado com alterações nas propriedades mecânicas e estruturais da parede vascular, levando à perda de elasticidade arterial e redução da complacência arterial. A complacência arterial pode ser medida por vários parâmetros, tais como, velocidade da onda de pulso, índice de aumento, e complacência arterial sistêmica.

Muitos estudos investigaram os efeitos da idade na rigidez arterial.^1 , 2^ A maioria sugere um aumento de VOP linear, relacionado à idade, e índice de aumento. Kim et al. demonstraram a relação entre idade e rigidez aórtica regional usando a RMC. Eles identificaram que a VOP regional era m ais alta na aorta torácica descendente e aumentada com a idade.^14^ Vários outros fatores e doenças também influenciaram a rigidez arterial, incluindo hipertensão, diabetes mellitus, hiperlipidemia, e tabagismo.^15 - 18^ Em nosso estudo, pacientes com VOP elevada também apresentaram maior prevalência de hipertensão, diabetes mellitus, e pressão arterial sistólica mais alta, se comparados àqueles com VOP não elevada, de forma consistente com relatórios anteriores.^15 , 16^

### Medição de rigidez aórtica

A VOP carótido-femoral por tonometria é geralmente o método de medição aceito para rigidez aórtica. Essa técnica é usada na maioria dos estudos clínicos como forte preditor de eventos cardiovasculares.^3 , 4^ Entretanto, esse método exige a medição presumida da distância aórtica da artéria carótida até a artéria femoral. A maioria dos estudos mediu a distância com fita sobre a superfície do corpo, levando a uma superestimativa da distância real percorrida pela onda de pulso.^3 , 4^

A medição de VOP usando a RMC é um dos métodos preferidos para se avaliar a rigidez aórtica, pois oferece alta resolução sem radiação ionizante. Além disso, a RMC pode medir a distância aórtica sem premissas geométricas, diferentemente da VOP carótido-femoral usando a tonometria. Os valores de VOP medidos por RMC em nossos estudos demonstraram imagens de alta qualidade com excelente reprodutibilidade, de forma consistente com um estudo anterior.^5^

### Envelhecimento e doença arterial coronariana

A idade é um fator de risco forte e independente para o desenvolvimento de aterosclerose coronária. Uma proporção significativa de pacientes idosos apresentou sintomas atípicos tais como fadiga, dispneia, e desconforto epigástrico. Testes ergométricos também são menos viáveis em pacientes idosos devido à capacidade de exercício mais baixa associada com a idade avançada e comorbidades, bem como normalidades no ECG da linha de base que limitam a avalição isquêmica. A RMC com estresse vasodilatador é uma modalidade não invasiva preferida usada para detectar isquemia miocárdica com viabilidade nessa população.

A isquemia miocárdica foi detectada em 36,1% dos pacientes como o preditor mais forte de MACE de análises multivariadas. Os achados ocorreram com relatórios anteriores.^9 , 19^ Evidências recentes sugerem que o RTG é um preditor poderoso de eventos cardiovasculares futuros em populações de pacientes abrangentes, incluindo adultos mais velhos.^20^ O RTG foi detectado em 14,8% dos pacientes. Considerando a pequena proporção de pacientes com histórico de IM (<2%), nossos resultados demonstraram ‘IM não reconhecido’ em pacientes idosos, compatível com dados anteriores.^21 , 22^

### VOP como preditor forte e independente nos idosos

A rigidez arterial é um preditor conhecido de eventos cardiovasculares. Vários estudos investigaram o valor prognóstico da rigidez arterial em adultos mais velhos aparentemente saudáveis,^3 , 4 , 23^ com certas inconsistências. Dois estudos encontraram associação entre rigidez arterial e eventos cardiovasculares, mas essa associação parecia ser limitada em outro estudo.^3 , 4 , 23^ Todos os estudos mediram a distância arterial para calcular a VOP pelo método da fita.^3 , 4 , 23^ Considerando os resultados inconsistentes anteriores e limitações de medição de VOP, nosso estudo buscou provar a hipótese e avaliar a VOP e a RMC-VP, que tem vantagens sobre a tonometria, conforme mencionado anteriormente.

Lui et al. relataram uma forte associação entre rigidez aórtica e biomarcadores de estresse miocárdico (peptídeo natriurético) e dano miocárdico (troponina cardíaca de alta sensibilidade) entre adultos mais velhos sem doença cardíaca.^24^ Também relatamos recentemente a associação da rigidez aórtica e a isquemia miocárdica, bem como o valor prognóstico da rigidez aórtica usando a RMC.^11 , 12^ Nossos resultados mostraram um aumento de quase duas vezes mais MACE entre os idosos com VOP elevada que também apresentou um valor prognóstico incremental sobre dados clínicos e variáveis de RMC, incluindo FEVE e isquemia miocárdica. O principal fator de MACE mais alto em nossos pacientes com VOP elevada foi um índice mais alto de acidente vascular isquêmico. Isso era consistente com estudos anteriores que demonstraram que a rigidez aórtica aumentava o risco de acidente vascular isquêmico (FC variando entre 2 e 4, dependendo do valor de corte da VOP), e a VOP continuava sendo significativamente preditiva de acidente vascular após a padronização para fatores de risco cardiovascular clássicos.^3 , 4^ Além disso, esses estudos incluíram adultos mais velhos e os idosos, assim como nosso estudo.^3 , 4^

### Utilidade da RMC para uma avaliação abrangente de DAC e rigidez aórtica

A utilidade da RMC para avaliar DAC é cada vez mais reconhecida, especialmente a RMC com perfusão por estresse vasodilatador, e avaliações de viabilidade pela técnica de RTG. Em nosso estudo, VOP e testes de estresse foram incorporados a um protocolo abrangente como uma vantagem exclusiva da RMC. A VOP foi medida durante o período de espera entre estudos de viabilidade e estresse, e a técnica sem prender a respiração se mostrou conveniente para os pacientes. As imagens de VOP foram adquiridas aproximadamente 10 minutos após a injeção de adenosina. A adenosina pode afetar a complacência arterial, mas isso não alterou as medições de VOP neste estudo, considerando sua meia vida muito rápida (<10 segundos).

### Terapia da rigidez aórtica

Para melhor evitar a ocorrência de eventos cardiovasculares, a modificação do estilo de vida bem como o tratamento anti-hipertensivo que reduza a rigidez aórtica devem ser considerados, ou seja, drogas que demonstraram sua eficácia na redução da VOP independentemente da redução da pressão arterial, incluindo os antagonistas do sistema renina-angiotensina-aldosterona e relaxamento de células do músculo liso por doadores de óxido nítrico ou moléculas relacionadas.^25 , 26^ Entretanto, grandes ensaios clínicos ainda precisam ser realizados para demonstrar que a prevenção de eventos cardiovasculares por esses agentes está associada à redução da rigidez aórtica, independentemente da redução da pressão arterial.^25 , 26^

## Limitações do estudo

Primeiramente, nosso estudo tinha uma população limitada, e algum nível de sobreajuste pode ter ocorrido durante as análises multivariadas; entretanto, a significância prognóstica da VOP foi demonstrada. Segundo, o estudo foi realizado em sujeitos asiáticos idosos, e a possibilidade de generalizar os dados para indivíduos mais jovens ou de outra etnia ainda não está certa. Terceiro, houve alguns valores de corte de VOP em adultos mais velhos/idosos sem doença cardiovascular de estudos anteriores (variando entre 9,5-13,2 m/s).^4 , 24^ Entretanto, nenhum nível de corte padrão foi determinado para VOP usando RMC para essa população. Por último, variações em frequências cardíacas poderiam ter resultado em formas de onda de velocidade ligeiramente diferentes entre ciclos cardíacos, resultando em erros de medição de VOP. Entretanto, um estudo anterior de validação de VOP medida por RMC determinou concordância entre medições de pressão intra-aórtica invasivas.^5^

## Conclusões

A rigidez aórtica avaliada por VOP baseada em RMC foi determinada como um marcador de risco forte e independente em pacientes idosos com suspeita de DAC ou DAC confirmada. Considerando o poder preditivo da VOP, a identificação de estratégias que possam evitar ou reduzir a rigidez pode ser importante na prevenção de eventos cardiovasculares. Esse aspecto exige investigações adicionais.

## * Material suplementar

Para informação adicional, por favor, clique aqui .
