# Supplementary material for: Valor Prognóstico de Rigidez Aórtica usando Ressonância Magnética Cardiovascular em Idosos com Suspeita ou Confirmação de Doença Arterial Coronariana
Source: Arq Bras Cardiol. 2022 May 4;118(5):961–71. [Article in Portuguese] doi: 10.36660/abc.20210452 (PMC9368868; doi:10.36660/abc.20210452)
Supplement: Translation

# Translation: Prognostic Value of Aortic Stiffness using Cardiovascular Magnetic Resonance in The Elderly with Known or Suspected Coronary Artery Disease

**Authors:** Yodying Kaolawanich, Thananya Boonyasirinant

**Affiliations:** 1 Thailand Division of Cardiology, Department of Medicine – Faculty of Medicine Siriraj Hospital, Mahidol University, Bangkok – Thailand

**Keywords:** Aortic Stiffness, Cardiovascular Magnetic Resonance, Coronary Artery Disease, Elderly, Prognosis

## Abstract

**Background:**

Aortic stiffness is established as a marker of cardiovascular disease. Cardiovascular magnetic resonance (CMR) provides a comprehensive assessment of aortic stiffness and myocardial ischemia in a single examination. However, prognostic data concerning aortic stiffness in elderly patients remain limited.

**Objective:**

To determine the prognostic value of aortic stiffness using CMR-based pulse wave velocity (PWV) in elderly patients with known or suspected coronary artery disease (CAD).

**Methods:**

This study enrolled consecutive patients aged >70 referred for adenosine stress perfusion CMR including PWV between 2010 and 2014. Patients were followed up for occurrence of major adverse cardiovascular events (MACE), including cardiac mortality, nonfatal myocardial infarction, hospitalization for heart failure, late revascularization (>180 days after CMR), and ischemic stroke. Univariable and multivariable analyses were performed to determine the predictors of MACE. A p-value of <0.05 is considered statistically significant.

**Results:**

Mean PWV was 13.98±9.00 m/s. After a median follow-up period of 59.6 months in 263 patients (55% female, 77±5 years), 61 MACE occurred. Patients with elevated PWV (>13.98 m/s) had significantly higher rates of MACE (HR 1.75; 95% CI 1.05-2.94; p=0.03) than those with non-elevated PWV (<13.98 m/s). Multivariate analysis demonstrated diastolic blood pressure, left ventricular ejection fraction (LVEF), myocardial ischemia, and elevated PWV as independent predictors for MACE (p<0.05 for all). PWV provided an incremental prognostic value over clinical data, LVEF, and ischemia (increased global chi-square=7.25, p=0.01).

**Conclusion:**

Aortic stiffness using CMR is a strong and independent predictor of cardiovascular events in elderly patients with known or suspected CAD.

## Introduction

Arterial stiffness increases with aging as an independent predictor of cardiovascular events, including mortality.^1 - 4^ There are several ways to measure arterial stiffness, including ultrasonography, carotid-femoral tonometer, and cardiovascular magnetic resonance (CMR). Measurement of aortic pulse wave velocity (PWV) by a tonometer has been extensively used. However, CMR is often the preferred method. CMR-based PWV measurements have been well validated (compared with invasive pressure recordings) with high reproducibility.^5^ Benefits of CMR include the provision of cross-sectional images covering the desired aortic length, high spatial resolution, and direct measurement of aortic length without geometric assumptions of the distance (in contrast to a tonometer), with no ionizing radiation.

Increased age is one of the most influential risk markers for cardiovascular disease (CVD), including coronary artery disease (CAD). CVD is responsible for over 80% of all deaths of individuals aged 65 or older in developed countries.^6^ Therefore, diagnosis and risk stratification of CAD in elderly patients is crucial. CMR provides a comprehensive assessment of CAD with very high accuracy.^7^ Moreover, adenosine stress CMR offers strong evidence for the prognosis of future cardiovascular events in patients with known or suspected CAD.^8^ Previous data indicated that stress CMR performed in ambulatory elderly is safe and well tolerated.^9 , 10^ CMR can assess PWV and perform a stress test in a single examination. We recently demonstrated the association of aortic stiffness and myocardial ischemia, as well as the prognostic value of aortic stiffness using CMR.^11 , 12^ Nevertheless, limited data exist concerning the prognosis of PWV by CMR in elderly patients.

This study objective aimed to determine the prognostic value of PWV in terms of major adverse cardiovascular events (MACE) in elderly patients with known or suspected CAD.

## Methods

### Study population

This study enrolled consecutive patients older than 70 years with known or suspected CAD who were referred for adenosine stress CMR from October 2010 to February 2014 to our outpatient center. In our institution, aortic stiffness using PWV has been routinely incorporated in comprehensive CMR protocol for CAD evaluation. Detailed medical history was collected on the day of the CMR study.

Exclusion criteria included (1) incomplete CMR examination, (2) contraindications to CMR (e.g., pacemaker) or adenosine (e.g., high-grade atrioventricular block), (3) unstable clinical condition, (4) patients with aortic diseases involving PWV measurement (e.g., an aortic aneurysm^13^ ), (5) poor CMR image quality, and (6) patients lacking follow-up data. Patients with a glomerular filtration rate of <30 ml/min/1.73 m^2^within 30 days before CMR were also excluded.

The institutional ethics committee approved this retrospective study and waived the need for additional written informed consent.

## CMR protocol ( Supplemental Materials )

### Cine, perfusion, and LGE image analyses ( Supplemental Materials )

#### PWV analysis11

Dedicated cardiovascular imaging software was applied for PWV analysis and performed independently from the perfusion study and LGE. Contours of mid-ascending and mid-descending thoracic aorta were drawn manually to achieve the flow (m/s) at both locations throughout all phases of the cardiac cycle. The corresponding flow-time curve was generated. Pulse wave arrival time was measured as the interception point of the linear extrapolation of the baseline and the steep early systolic stage, while aortic path length was determined by a multiplanar reconstruction of the axial half-Fourier acquisition from the steady-stage image. The reconstructed sagittal view of the path length was depicted as the centerline from the levels of the mid-ascending to the mid-descending thoracic aorta, corresponding to the same level obtained in VE-CMR.^11^

The PWV between the mid-ascending and mid-descending thoracic aorta was calculated as:


PWV=Δx/ΔT(m/s)

Where Δ x reflects the length of the aortic path between the mid-ascending and mid-descending thoracic aorta and Δ T represents the time delay between the arrival of the foot of the pulse wave at these two corresponding levels ( Supplemental Figure 1 ).

#### Intraobserver and interobserver variability of PWV measurement

Approximately 10% of the study cohort were randomly selected, using a Random Number Generator in Microsoft Excel, ver. 2016, to measure variability of the first observer 4 weeks after the initial analysis, and variability of the second independent observer, who was blinded to the initial results.

#### Clinical follow-up

Follow-up data were collected from clinical visits and medical records. Event adjudication was blinded to clinical and CMR data. Patients were followed up for MACE defined as composite outcomes for cardiac mortality, non-fatal myocardial infarction (MI), hospitalization for heart failure, late coronary revascularization (>180 days after CMR), and ischemic stroke. Need for revascularization therapy within 180 days after the CMR was considered to be triggered by the CMR results and therefore censored from analysis.

## Statistical analysis

Statistical analyses were performed using IBM SPSS Statistics for Windows, version 20.0 (IBM Corp., Armonk, NY, USA). Continuous variables with normal distribution were presented as mean ± standard deviation (SD), and continuous variables with non-normal distribution were presented as median and interquartile range. Normality distribution of the variables was examined by the Kolmogorov-Smirnov test. Categorical variables were presented as absolute numbers and percentages. Patients were divided into two groups based on their PWV values. Elevated PWV and non-elevated PWV groups used the mean PWV value of all patients as the cut-off level. Intraobserver and interobserver variability for PWV measurements were expressed as intraclass correlation coefficient (ICC), 95% confidence interval (CI) and bias ±2 SDs (for limits of agreement) using the Bland-Altman analysis.

Differences between patients with elevated and non-elevated PWV, as well as with and without MACE, were compared using the student’s unpaired t-test or Mann-Whitney U test for continuous variables, and the chi-square test or Fisher’s exact test for categorical variables, as appropriate.

Composite outcomes between both groups were estimated using the Kaplan-Meier method and compared with the log-rank test. To analyze the predictors of MACE, a Cox-regression analysis was performed to assess univariable predictors. Variables (baseline characteristics, medications at the time of CMR, and CMR parameters) with a p-value <0.05 in the univariable analysis were included for multivariable analysis using the ENTER method. A receiver operating characteristic (ROC) analysis was used to determine the best value of PWV predicting MACE.

To assess the incremental prognostic value of significant predictors, global chi-square values were calculated after adding predictors in the following order: clinical, LVEF, myocardial ischemia, and PWV.

All statistical tests were two-tailed, while all p-values of less than 0.05 were considered to indicate statistical significance.

## Results

### Patient characteristics

A total of 269 patients were enrolled, with two excluded due to having an aortic aneurysm and four excluded due to a loss of follow-up data. No patients were excluded because of poor image quality, and 263 were included in the final analysis. Mean age was 77.3±5.2 years. Table 1 summarizes patient clinical data. Two hundred and eight patients were referred for the first diagnosis of CAD. Fifty-five had been previously diagnosed with CAD, including 4 with previously documented MI. Overall, the study cohort had a mean LVEF of 68.1±15.1%. Myocardial ischemia was detected in 95 (36.1%) patients. Thirty-nine (14.8%) had LGE, and all showed a CAD pattern (subendocardial or transmural LGE). No patient presented an irregular heart rate (such as atrial fibrillation) during the PWV acquisition. Mean PWV was 13.98±9.00 m/s. History of hypertension, diabetes mellitus, and systolic blood pressure were independent predictors of elevated PWV (>13.98 m/s) ( Table 2 ).

**Table 1:** – Clinical Characteristics of Patients with and without Elevated PWV

	Total (n=263)	Elevated PWV (n=83)	Non-elevated PWV (n=180)	p-Value
Age (years)	77.3±5.2	77.9±5.1	77.1±5.2	0.19
Female	144 (54.8)	50 (60.2)	94 (52.2)	0.23
Body mass index (kg/m^2^)	26.3±4.1	26±4.1	26.4±4.2	0.49
Systolic BP (mmHg)	139.3±19.7	144.7±18.1	136.9±19.9	**0.003**
Diastolic BP (mmHg)	70.5±11.1	71.4±11.3	70.2±10.9	0.42
Heart rate (beats/minute)	76.6±13.9	76.6±15.5	76.6±13.2	0.99
**Clinical history**				
Hypertension	235 (89.4)	81 (97.6)	154 (85.6)	**0.01**
Diabetes mellitus	145 (55.1)	58 (69.9)	87 (48.3)	**0.001**
Hyperlipidemia	197 (74.9)	60 (72.3)	137 (76.1)	0.51
Coronary artery disease	55 (20.9)	20 (24.1)	35 (19.4)	0.39
Prior revascularization	12 (4.6)	4 (4.8)	8 (4.4)	0.89
Ischemic stroke	13 (4.9)	3 (3.6)	10 (5.6)	0.50
Cigarette smoker	28 (10.6)	7 (8.4)	21 (11.7)	0.43
**Medications**				
ACEI or ARB	130 (49.4)	43 (51.8)	87 (48.3)	0.60
Aspirin	134 (50.9)	45 (54.2)	89 (49.4)	0.47
Beta blocker	124 (47.2)	38 (45.8)	86 (47.8)	0.76
Calcium channel blocker	96 (36.5)	27 (32.5)	69 (38.3)	0.36
Statin	147 (55.9)	50 (60.2)	97 (53.9)	0.34
**CMR**				
LV mass (g)	84.3±24.5	83.6±25.5	84.7±24.0	0.74
LV ejection fraction (%)	68.1±15.1	69.8±14.1	67.3±15.5	0.21
Myocardial ischemia	95 (36.1)	28 (33.7)	67 (37.2)	0.58
Late gadolinium enhancement	39 (14.8)	15 (18.1)	24 (13.3)	0.32
PWV (m/s)	13.98±9.00	22.09±12.28	10.24±2.22	**<0.001**

*Values are number (percentages) or mean±SD. ***Bold*** values are <0.05. ACEI: angiotensin-converting enzyme inhibitor; ARB: angiotensin II receptor blocker; BP: blood pressure; CMR: cardiovascular magnetic resonance; LV: left ventricular; PWV: pulse wave velocity; SD: standard deviation.*

**Table 2:** – Predictors of Elevated PWV (>13.98 m/s)

	Univariate Analysis		Multivariate Analysis	

	HR (95% CI)	p-Value	HR (95% CI)	p-Value
Age (years)	1.03 (0.98. 1.09)	0.19		
Female	1.39 (0.82. 2.35)	0.23		
Body mass index (kg/m^2^)	0.98 (0.92. 1.04)	0.49		
Systolic BP (per 10 mmHg)	1.23 (1.07. 1.41)	**0.003**	1.23 (1.07. 1.42)	**0.01**
Diastolic BP (per 10 mmHg)	1.10 (0.87. 1.40)	0.42		
Hypertension	6.84 (1.58. 29.54)	**0.01**	6.06 (1.36. 26.97)	**0.02**
Diabetes mellitus	2.48 (1.43. 4.31)	**0.001**	2.09 (1.18. 3.70)	**0.01**
Hyperlipidemia	0.82 (0.45. 1.48)	0.51		
Coronary artery disease	1.32 (0.71. 2.54)	0.39		
Prior revascularization	1.09 (0.32. 3.72)	0.89		
Ischemic stroke	0.64 (0.17. 2.38)	0.50		
Cigarette smoker	0.70 (0.28. 1.71)	0.43		
ACEI or ARB	1.15 (0.68. 1.93)	0.60		
Aspirin	1.21 (0.72. 2.04)	0.47		
Beta blocker	0.92 (0.55. 1.56)	0.76		
Calcium channel blocker	0.78 (0.45. 1.34)	0.36		
Statin	1.30 (0.76. 2.20)	0.34		
LV mass (g)	0.99 (0.98. 1.01)	0.74		
LV ejection fraction (per 10%)	1.13 (0.94. 1.35)	0.21		
Myocardial ischemia	0.86 (0.50. 1.48)	0.58		
Late gadolinium enhancement	1.43 (0.71. 2.90)	0.32		

****Bold*** values are <0.05. ACEI: angiotensin-converting enzyme inhibitor; ARB: angiotensin II receptor blocker; BP: blood pressure; CMR: cardiovascular magnetic resonance; LV: left ventricular; PWV: pulse wave velocity; CI: confidence interval; HR: hazard ratio.*

#### Intraobserver and interobserver variability for PWV measurement

There was less intraobserver and interobserver variability for PWV measurements by VE-CMR ( Figure 1 ). For the 30 randomly selected patients, mean PWV±SD values were 9.88±2.73 m/s and 9.87±2.59 m/s for the first observer in the initial analysis and 4 weeks later, respectively, and 9.94±2.67 m/s for the second observer in the initial analysis. There was no significant bias (mean difference for intraobserver=0.01±0.49 m/s, p=0.98 and for interobserver=-0.03±0.35 m/s, p=0.93) ( Figure 1B and 1D , respectively).

**Figure 1:** – Intraobserver and interobserver variability of PWV measurements. Intraclass correlation (A for intraobserver and C for interobserver) and Bland-Altman plot (B for intraobserver and D for interobserver). ICC: intraclass correlation coefficient; PWV: pulse wave velocity.

#### Primary outcome: MACE

During the median follow-up period of 59.6 months (interquartile range: 36.6, 68.2 months), 61 MACE occurred. Clinical characteristics including CMR variables of patients with and without MACE are shown in Supplemental Table 1 . Patients with MACE had significantly lower diastolic blood pressure, higher LV mass, lower LVEF, and a higher prevalence of ischemia and LGE.

Table 3 demonstrates cardiovascular events in the study cohort. Figure 2A shows the Kaplan-Meier curves of patients with and without elevated PWV. Patients with elevated PWV had significantly higher rates of MACE than those with non-elevated PWV. Figure 2B demonstrates the Kaplan-Meier curves stratified by the presence of ischemia with and without elevated PWV. Patients with non-elevated PWV and negative ischemia had the best outcome, while the patients with elevated PWV and positive ischemia had the worst outcome. Note that the patients with non-elevated PWV and positive ischemia had no difference in the rate of MACE compared to the patients with elevated PWV and negative ischemia (HR 2.03, 95% CI 0.89-4.63, p=0.09).

**Table 3:** – Cardiovascular Events

	Total (n=263)	Elevated PWV (n=83)	Non-elevated PWV (n=180)	HR (95% CI)	p-Value
MACE^a^	61 (23.2)	24 (28.9)	37 (20.6)	1.75 (1.05. 2.94)	**0.03**
Cardiac mortality	5 (1.9)	2 (2.4)	3 (1.7)	1.68 (0.28. 10.07)	0.57
Nonfatal myocardial infarction	24 (9.1)	9 (10.8)	15 (8.3)	1.60 (0.70. 3.67)	0.27
Hospitalization for heart failure	36 (13.7)	15 (18.1)	21 (11.7)	1.94 (0.99. 3.81)	0.05
Late coronary revascularization	16 (6.1)	5 (6.0)	11 (6.1)	1.17 (0.41. 3.39)	0.77
Ischemic stroke	11 (4.2)	7 (8.4)	4 (2.2)	5.04 (1.47. 17.32)	**0.01**

*MACE = composite outcomes of cardiac mortality, nonfatal myocardial infarction, hospitalized for heart failure, late coronary revascularization, and ischemic stroke.^a^Nineteen patients had more than one event. Values are numbers (percentages). ***Bold*** values are <0.05. CI: confidence interval; HR: hazard ratio; MACE: major adverse cardiovascular events; PWV: pulse wave velocity.*

**Figure 2:** – Kaplan-Meier curves for MACE. For the whole cohort, patients with elevated PWV had significantly higher rates of MACE than those with non-elevated PWV (Figure 2A). Figure 2B demonstrates the Kaplan-Meier curves stratified by the presence of ischemia with and without elevated PWV. MACE: major cardiovascular events; PWV: pulse wave velocity.

A ROC curve ( Figure 3 ) demonstrated the best value of PWV of 11.16 m/s to predict MACE with a sensitivity of 71% and specificity of 50%.

**Figure 3:** – A ROC curve demonstrates the best value of PWV to predict MACE. MACE: major cardiovascular events; PWV: pulse wave velocity; ROC: receiver operating characteristic.

Univariate and multivariate analyses for prediction of MACE are shown in Table 4 . Univariate analysis demonstrated diastolic blood pressure, history of CAD, LV mass, LVEF, ischemia, LGE, and elevated PWV as predictors. Multivariate analysis revealed diastolic blood pressure, LVEF, ischemia, and elevated PWV as independent predictors for MACE.

**Table 4:** – Predictors of MACE

	Univariate Analysis		Multivariate Analysis	

	HR (95% CI)	p-Value	HR (95% CI)	p-Value
Age (years)	1.03 (0.98. 1.08)	0.29	1.02 (0.97. 1.08)	0.43
Female	1.01 (0.61. 1.67)	0.98		
Body mass index (kg/m^2^)	0.95 (0.89. 1.01)	0.08		
Systolic BP (per 10 mmHg)	0.89 (0.78. 1.02)	0.10		
Diastolic BP (per 10 mmHg)	0.75 (0.59. 0.96)	**0.01**	0.76 (0.59. 0.97)	**0.03**
Hypertension	1.49 (0.60. 3.71)	0.40		
Diabetes mellitus	1.17 (0.70. 1.94)	0.55		
Hyperlipidemia	1.37 (0.73. 2.58)	0.33		
Coronary artery disease	1.80 (1.02. 3.17)	**0.04**	1.25 (0.69. 2.26)	0.47
Prior revascularization	1.56 (0.62. 3.89)	0.35		
Ischemic stroke	0.63 (0.15. 2.58)	0.52		
Cigarette smoker	1.37 (0.65. 2.88)	0.41		
ACEI or ARB	1.39 (0.84. 2.31)	0.20		
Aspirin	1.35 (0.81. 2.25)	0.25		
Beta blocker	1.15 (0.69. 1.89)	0.60		
Calcium channel blocker	0.88 (0.52. 1.49)	0.62		
Statin	0.99 (0.60. 1.64)	0.97		
LV mass (g)	1.02 (1.01. 1.03)	**0.001**	1.01 (0.99. 1.02)	0.41
LV ejection fraction (per 10%)	0.75 (0.65. 0.86)	**<0.001**	0.84 (0.70. 0.99)	**0.04**
Myocardial ischemia	3.10 (1.86. 5.18)	**<0.001**	2.26 (1.23. 4.14)	**0.01**
Late gadolinium enhancement	2.30 (1.27. 4.19)	**0.01**	1.08 (0.55. 2.12)	0.8
Elevated PWV (>13.98 m/s)	1.75 (1.05. 2.94)	**0.03**	1.99 (1.17. 3.40)	**0.01**

****Bold*** values are <0.05. ACEI: angiotensin-converting enzyme inhibitor; ARB: angiotensin II receptor blocker; BP: blood pressure; CMR: cardiovascular magnetic resonance; LV: left ventricular; PWV: pulse wave velocity; CI: confidence interval; HR: hazard ratio; MACE: major adverse cardiovascular events; PWV: pulse wave velocity.*

#### Incremental prognostic value of PWV

Table 5 shows an incremental prognostic value of clinical and CMR data for the prediction of MACE. When the prognosis was assessed in a hierarchical manner (clinical only, clinical+LVEF, clinical+LVEF+myocardial ischemia, and clinical+LVEF+myocardial ischemia+PWV), LVEF and ischemia provided an incremental prognostic value over clinical data. PWV added a further incremental prognostic value over LVEF, and ischemia.

**Table 5:** – Incremental Prognostic Value of PWV for MACE

	Global 𝛘^2^	Increase in 𝛘^2^	p-value
Clinical	10.19	–	–
Clinical + LVEF	27.11	14.17	**<0.001**
Clinical + LVEF + Myocardial ischemia	38.55	10.02	**0.01**
Clinical + LVEF + Myocardial ischemia + PWV	45.21	7.25	**0.01**

****Bold*** values are <0.05. Clinical=age, female gender, diastolic blood pressure, and history of coronary artery disease. 𝛘^2^= chi-square. LVEF: left ventricular ejection fraction; PWV: pulse wave velocity.*

## Discussion

Results demonstrated aortic stiffness, assessed by VE-CMR, as a strong predictor of MACE, regardless of traditional risk factors, cardiac function, myocardial ischemia, and LGE in elderly patients with known or suspected CAD. PWV also provided an incremental prognostic value over clinical data, LVEF, and myocardial ischemia.

### Aging and vascular change

Vascular aging is associated with changes in the mechanical and structural properties of the vascular wall, leading to a loss of arterial elasticity and reduced arterial compliance. Arterial compliance can be measured by different parameters, such as pulse wave velocity, augmentation index, and systemic arterial compliance.

Many studies have investigated the effects of age on arterial stiffness,^1 , 2^ with most suggesting a linear, age-related increase in PWV and augmentation index. Kim et al. demonstrated the relationship between age and regional aortic stiffness using CMR. They found that the regional PWV was highest in the descending thoracic aorta and increased with age.^14^ Several other factors and diseases also influence arterial stiffness, including hypertension, diabetes mellitus, hyperlipidemia, and smoking^15 - 18^ In our study, patients with elevated PWV also showed an increased prevalence of hypertension, diabetes mellitus, and higher systolic blood pressure when compared to those with non-elevated PWV, which is consistent with previous reports.^15 , 16^

### Measurement of aortic stiffness

Carotid-femoral PWV using a tonometer is the generally accepted measurement method for aortic stiffness. This technique is used in most clinical studies as a strong predictor of cardiovascular events.^3 , 4^ However, this method requires the assumed measurement of the aortic distance from the carotid to femoral arteries. Most studies measured this distance with tape over the surface of the body, leading to an overestimation of the real distance traveled by the pulse wave.^3 , 4^

PWV measurement using CMR is one of the preferred methods to evaluate aortic stiffness, providing high resolution without ionizing radiation. Moreover, CMR can measure aortic distance without geometrical assumptions, unlike carotid-femoral PWV using tonometry. PWV values measured by CMR in our study demonstrated high-quality images with excellent reproducibility, which is consistent with a previous study.^5^

### Aging and coronary artery disease

Age is a strong and independent risk factor for the development of coronary atherosclerosis. A significant proportion of elderly patients presented atypical symptoms, such as fatigue, dyspnea, and epigastric discomfort. Exercise testing is also less feasible in elderly patients due to the lower exercise capacity associated with advanced age and co-morbidities, as well as baseline ECG abnormalities that limit ischemic assessments. Vasodilatory stress CMR is a preferred non-invasive modality used to detect myocardial ischemia with viability in this population.

Myocardial ischemia was detected in 36.1% of the patients as the strongest predictor of MACE from a multivariate analysis. Findings concurred with previous reports.^9 , 19^ Recent evidence suggests that LGE is a powerful predictor of future cardiovascular events in wide-ranging patient populations, including older adults.^20^ LGE was detected in 14.8% of the patients. Given the very small proportion of patients with a history of MI (<2%), our results demonstrated ‘unrecognized MI’ in elderly patients, which is compatible with previous data.^21 , 22^

### PWV as a strong and independent prognosticator in the elderly

Arterial stiffness is a well-known predictor of cardiovascular events. Several studies investigated the prognostic value of arterial stiffness in apparently healthy older adults,^3 , 4 , 23^ with certain inconsistencies. Two studies found an association between arterial stiffness and cardiovascular events, but this association appeared to be limited in another study.^3 , 4 , 23^ All studies measured arterial distance to calculate PWV by the tape method.^3 , 4 , 23^ Given previous inconsistent results and limitations of PWV measurement, our study sought to prove the hypothesis and assess PWV by VE-CMR, which has advantages over tonometry, as previously mentioned.

Lui et al. reported a strong association between aortic stiffness and biomarkers of both myocardial stress (natriuretic peptide) and damage (high-sensitivity cardiac troponin-T) among older adults without cardiac disease.^24^ Our research team also recently reported the association of aortic stiffness and myocardial ischemia as well as the prognostic value of aortic stiffness using CMR.^11 , 12^ Our results showed an almost 2-fold increase in MACE among the elderly with elevated PWV, which also provided an incremental prognostic value over clinical data and CMR variables, including LVEF and myocardial ischemia. The main driver of higher MACE in our patients with elevated PWV was a higher rate of ischemic stroke. This was consistent with previous studies that aortic stiffness increased the risk of ischemic stroke (HR ranged from^2 - 4^ , depending on the cutoff of PWV), while PWV remained significantly predictive of stroke after adjustment for classical cardiovascular risk factors.^3 , 4^ Additionally, these studies included older adults and the elderly, similar to our study.^3 , 4^

### Usefulness of CMR for a comprehensive assessment of CAD and aortic stiffness

The use of CMR to evaluate CAD is being increasingly recognized, particularly as vasodilator stress perfusion CMR and viability assessments by LGE technique. In our study, PWV and stress tests were incorporated into a comprehensive protocol as the unique advantage of CMR. PWV was measured during the waiting period between the stress and viability studies, and the non-breath-hold technique proved convenient for patients. PWV images were acquired approximately 10 minutes after adenosine injection. Adenosine may affect arterial compliance, but this did not alter PWV measurements in this study, given its very rapid half-life (<10 seconds).

### Therapy of aortic stiffness

To better prevent the occurrence of cardiovascular events, lifestyle modification, as well as antihypertensive treatment that reduce aortic stiffness should be considered, i.e., drugs that have demonstrated their efficacy in reducing PWV regardless of the reduction in blood pressure, including the renin-angiotensin-aldosterone-system antagonists and smooth muscle cell relaxation by nitric oxide donors or related molecules.^25 , 26^ However, large clinical trials have yet to be performed to demonstrate that the prevention of cardiovascular events by these agents is associated with the reduction in aortic stiffness, regardless of blood pressure reduction.^25 , 26^

## Study limitations

First, our study had a limited population, and some degree of overfitting may have occurred during the multivariate analyses; however, the prognostic significance of PWV was demonstrated. Second, the study was conducted on elderly Asian subjects, and data generalizability to younger individuals or other ethnicities remains uncertain. Third, there were some PWV cutoff values in older adults/elderly without cardiovascular disease from prior studies (ranged 9.5-13.2 m/s).^4 , 24^ However, no standard cutoff level was determined for PWV using CMR for this population. Finally, variations in heart rates could have resulted in slightly different velocity waveforms between cardiac cycles, resulting in PWV measurement errors. However, a previous validation study of PWV measured by CMR determined agreement between invasive intra-aortic pressure measurements.^5^

## Conclusions

Aortic stiffness assessed by CMR-based PWV was determined as a strong and independent risk marker in elderly patients with known or suspected CAD. Given the predictive power of PWV, identifying strategies that can prevent or reduce stiffening may be important in the prevention of cardiovascular events. This aspect requires further investigation.

## * Supplemental Materials

For additional information, please click here .
